# Different Renal Function Patterns in Patients With Acute Heart Failure: Relationship With Outcome and Congestion

**DOI:** 10.3389/fcvm.2022.779828

**Published:** 2022-03-07

**Authors:** Alberto Palazzuoli, Federico Crescenzi, Lorenzo Luschi, Angelica Brazzi, Mauro Feola, Arianna Rossi, Antonio Pagliaro, Nicolò Ghionzoli, Gaetano Ruocco

**Affiliations:** ^1^Cardiovascular Diseases Unit, Department of Medical Sciences, Le Scotte Hospital, University of Siena, Siena, Italy; ^2^Department of Statistics, University of Florence, Florence, Italy; ^3^Cardiology Section, Regina Montis Regalis Hospital, ASL-CN1, Cuneo, Italy; ^4^Department of Geriatrics, University of Turin, Turin, Italy; ^5^Cardiology Unit, Le Scotte Hospital, University of Siena, Siena, Italy; ^6^Cardiology Unit, “Riuniti of Valdichiana” Hospital, Usl-Sudest Toscana, Montepulciano, Italy

**Keywords:** acute heart failure (AHF), renal dysfunction, congestion, outcome, worsening renal function (WRF)

## Abstract

**Background:**

The role of worsening renal function during acute heart failure (AHF) hospitalization is still debated. Very few studies have extensively evaluated the renal function (RF) trend during hospitalization by repetitive measurements.

**Objectives:**

To investigate the prognostic relevance of different RF trajectories together with the congestion status in hospitalized patients.

**Methods:**

This is a *post hoc* analysis of a multi-center study including 467 patients admitted with AHF who were screened for the Diur-AHF Trial. We recognized five main RF trajectories based on serum creatinine and estimated glomerular filtration rate (eGFR) behavior. According to the RF trajectories our sample was divided into 1-stable (S), 2-transient improvement (TI), 3-permanent improvement (PI), 4-transient worsening (TW), and 5-persistent worsening (PW). The primary outcome was the combined endpoint of 180 days including all causes of mortality and re-hospitalization.

**Results:**

We recruited 467 subjects with a mean congestion score of 3.5±1.08 and a median creatinine value of 1.28 (1.00–1.70) mg/dl, eGFR 50 (37–65) ml/min/m^2^ and NTpro B-type natriuretic peptide (BNP) 7,000 (4,200–11,700) pg/ml. A univariate analysis of the RF pattern demonstrated that TI and PW patterns were significantly related to poor prognosis [HR: 2.71 (1.81–4.05); *p* < 0.001; HR: 1.68 (1.15–2.45); *p* = 0.007, respectively]. Conversely, the TW pattern showed a significantly protective effect on outcome [HR:0.34 (0.19–0.60); *p* < 0.001]. Persistence of congestion and BNP reduction ≥ 30% were significantly related to clinical outcome at univariate analysis [HR: 2.41 (1.81–3.21); *p* < 0.001 and HR:0.47 (0.35–0.67); *p* < 0.001]. A multivariable analysis confirmed the independently prognostic role of TI, PW patterns, persistence of congestion, and reduced BNP decrease at discharge.

**Conclusions:**

Various RF patterns during AHF hospitalization are associated with different risk(s). PW and TI appear to be the two trajectories related to worse outcome. Current findings confirm the importance of RF evaluation during and after hospitalization.

## Introduction

Renal function (RF) deterioration occurring in acute heart failure (AHF) is one of the most important features during hospitalization with several repercussions on treatment and prognosis. Interventional trials reported a wide percentage of worsening renal function (WRF) ranging from 20 to 40% with a different prognostic impact (1–3). The unreadable relationship existing between WRF appearance and heart failure (HF) outcome is currently under debate, and the question of whether renal function impairment is just a marker or a true component of HF severity remains unanswered (4). Several definitions have been suggested to elucidate the exact role and prevalence of renal dysfunction in the setting of HF (5, 6). Some studies report that WRF is a detrimental factor in HF deterioration and outcome. Other authors described this status as a simple condition linked to decongestion therapy and the high loop diuretic amount administered during the acute phase (7–10). Alternatively, an increased neurohormonal overdrive and altered renal blood flow redistribution may be implicated. Predisposing common risk factors, such as diabetes, smoking, metabolic disorders, and hypertension, could amplify the cardiac and kidney atherosclerosis process and increase neurohormonal overdrive, leading to a final systemic and renal hemodynamic derangement (11). This assessment should be considered during AHF hospitalization and should be interpreted by looking at both the individual patients and their primary cardiac and renal disorder. The bidirectional nature and the specific mechanisms related to the vicious circle of coexisting cardiac and renal deterioration still need to be completely explained. Indeed, the current cardio-renal syndrome type 1 (CRS-1) update only recognizes the primary and secondary organ damage (12, 13). Unfortunately, this definition does not yet provide an extensive interplay regarding the pathophysiological cross-talk, including predisposing factors, haemodynamic derangement, and preliminary kidney condition, implicated in its occurrence and organ deterioration (14). Notably, several studies have shown significant fluctuation of renal function during hospitalization that likely reflects the diversity of the RF pattern in these patients (15–18). Accordingly, a more complete mechanistic approach should include serial measurement, a basal renal evaluation at admission, a serial assessment during hospitalization, and monitoring after discharge (5, 19). Thus, the identification of specific renal trajectories occurring during acute treatment appear mandatory in order to better understand the individual heterogeneity of renal pattern and the related risk. Therefore, a better recognition of RF changes over hospitalization could improve insights into the assessment existing among HF conditions, congestion, and outcome. Notably, we divided our patients according to RF fluctuations during the hospitalization period and we recognized 5 main subtypes. Then, we evaluated each subtypes in relation to the prognosis during a mean follow up period of 6 months.

## Methods

### Study Design

This is a retrospective *post hoc* analysis of a multi-center study including 467 patients admitted with AHF who were screened for the Diur-AHF Trial (20) and enrolled in both the Cardiovascular Diseases Unit of Internal Medicine Department and Cardiology Unit of Siena Hospital and the Cardiology Section of Regina Montis Regalis Hospital of Mondovì (Cuneo). The patients screened were over the age of 18 years and were admitted with dyspnoea, evidence of volume overload, and/or clinical signs of HF (peripheral edema, rales, third heart sound, jugular turgor, lung congestion on chest X-ray) in whom a diagnosis of AHF was confirmed by chest X-ray and/or elevated (>1,500 pg/ml) levels of Amino-terminal (NT) pro-B-type natriuretic peptide (BNP). The excluded patients were those with end-stage (serum creatinine levels > 4.0 mg/dL) renal disease or the need for renal replacement therapy (dialysis or ultrafiltration), a recent myocardial infarction (within 30 days of screening), a systolic blood pressure <80 mm Hg, or the needing of vasoactive or inotropic drugs infusion during hospitalization. We did not screen patients with known liver or neoplastic disease or concurrent infective disease. All patients gave their written informed consent. This study was approved by the local ethics committee of Siena Hospital (C.E.A.V.S.E.).

### Laboratory Analysis

Renal function parameters, including creatinine, estimated glomerular filtration rate (eGFR), and blood urea nitrogen (BUN), were measured from blood samples taken at admission, 3 days after admission, and before discharge. Renal function parameters were monitored every 48 h. The eGFR was calculated using the four-variable Modification of Diet in Renal Disease (MDRD) formula. Chronic Kidney Disease (CKD) was defined by creatinine > 1.2 mg/dl and/or eGFR <60 ml/min/1.73 m^2^ before admission (21, 22). A rise in serum creatinine ≥0.3 mg/dl or eGFR reduction ≥ 20% were used according to conventional criteria to define worsening renal function (WRF). NTpro BNP was also measured within 24 h of hospital admission and before discharge.

### RF Pattern Definition

**Figure 1:**
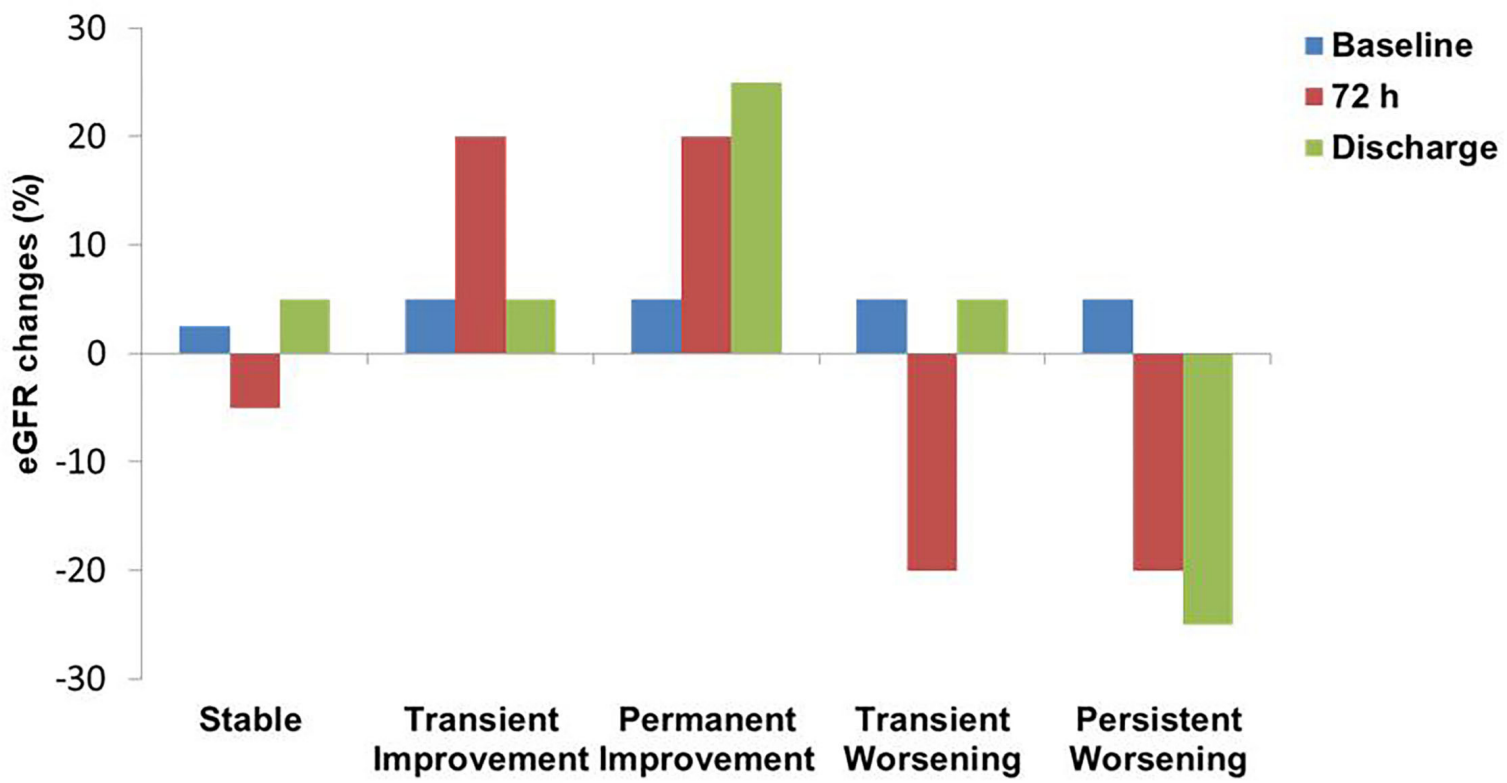
Renal function patterns definition according to eGFR fluctuations. eGFR, estimated Glomerular filtration rate.

We identified 5 main trajectories based on changes in creatinine and GFR summarized in Figure 1:

1. Stable pattern (S) identifies patients with no substantial or minimal differences in renal function;
2. Bump pattern: a transient improvement (TI) of 0.2 or > 20% in creatinine and GFR, respectively, followed by a subsequent deterioration;
3. Permanent improvement (PI) characterized by gradual amelioration of creatinine and GFR during hospitalization;
4. Transient worsening (TW) of Renal function followed by an amelioration before discharge;
5. Persistent worsening (PW) due to progressive impairment of Creatinine and GFR from admission to discharge.

### Clinical Assessment and Evaluation of Congestion

All these patterns were compared with a clinical evaluation of congestion at admission and before discharge, grading congestion by the assessment of the following clinical signs: pulmonary rales, third heart sound, jugular venous distention, peripheral oedema, hepatomegaly, and dyspnoea at rest or orthopnoea for a total of maximum 6 points based on Gheorghiade criteria (23). Persistence of congestion was defined as the persistence of 3 signs of congestion at discharge or less if the patients did not achieve the complete resolution of two or more clinical signs of HF at discharge.

### End Points

(1) To discern among different RF patterns during hospitalization for Acute decompensated Heart failure (ADHF); (2) To evaluate whether various RF subtypes were associated with different outcomes in terms of combined endpoint of mortality and HF rehospitalization during a 180-day follow-up.

### Follow-Up

Patients were followed up for 180 days after discharge with clinical visits and telephone contacts. The primary outcome of interest was a composite of all-cause mortality (ACM) or cardiovascular (CV) re-hospitalization.

### Statistical Analysis

Categorical data are presented as numbers and percentages and were analyzed with chi-square test. Particularly, normally distributed continuous data were presented as mean ± SD and non-normally distributed continuous variables were presented as median and interquartile range [IQR]. Patients with AHF were grouped by phenotypes according to WRF subtypes, significant decongestion during hospitalization, and CKD. Differences in baseline characteristics for continuous variables were evaluated using appropriate procedures like the *T*-Student test or Mann Whitney test if two groups were compared. Differences among more than two groups were analyzed with ANOVA and Kruskal-Wallis tests. Kaplan-Meier curves with the log-rank statistics were used to illustrate event rates at the time point of interest. Different multivariable Cox proportional hazard regression models were used to investigate the relationship between WRF subtypes and outcomes. Multivariable models were adjusted for clinical variables of interest (age, gender, hypertension, diabetes, dyslipidemia, coronary artery disease, history of HF, and LVEF) chosen prospectively a priori. Estimates are presented as hazard ratios (HR) with 95% CI. We considered statistically significant results associated with a *p* ≤ 0.05. We used the SPSS software (version 20.0) for all analyses.

## Results

The initial study population consisted of 499 patients enrolled in three different hospitals (the Cardiology Unit of Mondovi Hospital, the Cardiology Unit, and Internal Medicine Unit of Le Scotte Hospital, Siena) with a primary diagnosis of AHF. Twelve patients were lost during the follow-up period and 20 patients were not included for lack of complete clinical and laboratory examination. Among the remaining 467 subjects 55.5% were males with a median age of 78 (67–84) years. Median LVEF was 38% (29–45), mean congestion score was 3.5±1.08, and median creatinine value was 1.28 (1.00–1.70) mg/dl, median eGFR and NTproBNP were 50 (37–65) ml/min /m^2^ and 7,000 (4,200–11,700) pg/ml, respectively. Complete clinical and laboratory characteristics were divided between patients with adverse events occurrence and those patients free of adverse events are described in Table 1.

**Table 1:** Clinical and laboratory characteristics according to adverse events development or not.

**Variables**	Patients without adverse events^*^(*n*. 275)	Patients with adverse events occurrence^*^ **(*n*. 192)**	***p*-value**
Age (years)	78 [67–84]	78 [67–85]	0.785
Gender male (%)	62.2	45.8	<0.001
**CV risk factors (%)**			
Hypertension	63.6	72.9	0.035
Diabetes	31.4	37.5	0.170
Dyslipidemia	21.1	23.6	0.528
CAD	48.0	66.7	<0.001
CKD	48.0	59.9	0.011
Atrial fibrillation	17.5	35.4	<0.001
**HF etiology**			
Hypertensive	50.9	42.2	0.063
Ischemic	20.4	25.0	0.236
Valvular	16.7	21.9	0.162
Primitive	12.0	10.9	0.724
**Echocardiography**			
LVEF (%)	40 [30–45]	35 [25–45]	0.078
LVEDD (mm)	54 [48–59]	55 [48–61]	0.194
LVESD (mm)	38 [32–45]	40 [33–47]	0.127
Basal RVEDD (mm)	37 [36–43]	38 [36–44]	0.161
TAPSE (mm)	20 [18–22]	20 [16–22]	0.025
IVC (mm)	22 [20–23]	22 [20–24]	0.203
PASP (mmHg)	40 [35–50]	45 [35–50]	0.464
E/e'	15 [13–16]	15 [14–16]	0.724
Systolic arterial pressure	134 [126–140]	135 [126–145]	0.402
Admission congestion score	3.3 [±1.1]	3.8 [±0.9]	<0.001
Discharge congestion score	0.8 [±1.1]	1.9 [±1.0]	<0.001
Admission serum creatinine (mg/dL)	1.21[0.98–1.62]	1.43 [1.00–1.86]	0.007
Discharge serum creatinine (mg/dL)	1.25 [1.00–1.66]	1.49 [1.08–2.00]	<0.001
Admission eGFR (mL/min/m^2^)	52 [38–66]	48 [33–63]	0.049
Discharge eGFR (mL/min/m^2^)	52 [36–69]	45 [30–61]	0.002
In-hospital IV mean daily furosemide dosage (mg/die)	100 [80–120]	125 [120–150]	<0.001
Admission NTproBNP (pg/mL)	7,100 [4,188–11,297]	6,837 [4,202–13,269]	0.784
Discharge NTproBNP (pg/mL)	2,664 [1,178–6,021]	4,627 [1,906–7,888]	<0.001
Previous CHF (%)	52.7	61.5	0.061
ICD (%)	12.4	12.0	0.901
**Home therapy (%)**			
Loop diuretics	53.8	60.9	0.127
ACEis/ARBs/ARNI	81.8	58.9	<0.001
Beta Blockers	82.2	61.5	<0.001
MRAs	6.9	13.5	0.017
Digoxin	0.0	18.8	<0.001
Ivabradin	12.7	24.0	0.002

*ACEis, Angiotensin Converting Enzyme Inhibitors; ARBs, Angiotensin receptor blockers; ARNI, Angiontensin Receptor Neprilysin Inhibitor; CV, Cardiovascular; CHF, Chronic heart failure; CKD, Chronic Kidney Disease; CAD, Coronary artery disease; eGFR, estimated Glomerular filtration rate; HF, Heart Failure; ICD, Implantable cardiac defibrillator; IV, Intravenous; IVC, Inferior Cave Vein; LVEDD, Center ventricular end-diastolic diameter; LVEF, Center ventricular ejection fraction; LVESD, Center ventricular end-systolic diameter; MRAs, Mineralocorticoid receptor antagonists; NTproBNP, Aminoterminal pro B-type natriuretic peptide; PASP, Pulmonary artery systolic pressure; RVEDD, Right ventricular end-diastolic diameter; TAPSE, Tricuspid anular plase systolic excursion*.

* *Events are defined as the composite of all-cause mortality (ACM) or cardiovascular (CV) re-hospitalization*.

### Baseline Characteristics

Baseline characteristics of different trajectories are shown in Table 2. Overall, there are no significant differences among different trajectories regarding risk factor prevalence, except for dyslipidaemia, which is significantly prevalent in patients with PW compared to TW, PI, TI, and stable S (27.5 vs. 14.4, vs. 12.7 vs. 24.6 vs. 26.6%, respectively; *p* = 0.039). The admission congestion score resulted significantly higher in S, TI, and PW groups with respect to PI and TW groups [3.63 (±1.02) vs. 3.53 (±1.05) vs. 3.80 (±1.10) vs. 3.27 (±1.10) vs. 3.13 (±1.05), respectively; *p* < 0.001]. Echocardiographic measurements of right ventricle, pulmonary pressures, and left ventricle filling pressures were not different among the groups. CKD was more prevalent in patients who experienced renal function in-hospital improvement (group TI and PI) with respect to S, TW, and PW groups (71.9 and 71.4% vs. 37.3%, 57.1 and 50.5% respectively; *p* < 0.001). Conversely, patients of S, TW, and PW groups demonstrated better baseline renal function variables (creatinine and eGFR) as reported in Table 2.

**Table 2:** Differences in clinical and laboratory characteristics according to renal function patterns.

	**Renal function patterns (n. of patients)**					
**Variables**	**S (158)**	**TI (57)**	**PI (63)**	**TW (98)**	**PW (91)**	***p*–value**
Age (years)	80 [69–84]	77 [65–84]	81 [74–88]	77 [64–84]	75 [66–81]	0.001
Gender Male (%)	51.3	36.8	61.9	62.2	62.6	0.008
**CV risk factors (%)**						
Hypertension	63.3	68.4	65.1	67.3	75.8	0.362
Diabetes	32.9	42.9	33.3	33.7	30.8	0.648
Dyslipidemia	26.6	24.6	12.7	14.4	27.5	0.039
CAD	55.7	61.4	57.1	50.5	57.1	0.704
CKD	37.3	71.9	71.4	57.1	50.5	<0.001
Atrial fibrillation	17.1	36.8	30.2	21.4	30.8	0.01
**Echocardiography**						
LVEF (%)	35 [30–50]	40 [30–45]	40 [27–50]	39 [29–45]	35 [25–45]	0.467
LVEDD (mm)	55 [49–59]	54 [48–59]	53 [45–58]	54 [47–60]	55 [49–63]	0.279
LVESD (mm)	39 [33–45]	39 [33–44]	39 [31–43]	38 [31–46]	40 [34–48]	0.204
Basal RVEDD (mm)	38 [36–44]	37 [36–43]	37 [36–43]	36 [35–40]	38 [35–44]	0.283
TAPSE (mm)	20 [18–22]	20 [18–22]	20 [16–22]	21 [19–22]	20 [16–22]	0.460
IVC (mm)	22 [20–23]	22 [20–23]	22 [20–24]	22 [20–23]	22 [21–24]	0.223
PASP (mmHg)	40 [35–50]	45 [35–45]	40 [35–50]	40 [35–45]	45 [35–50]	0.169
E/e'	15 [14–17]	14 [12–15]	14 [14–16]	14 [12–16]	15 [14–16]	0.090
Systolic arterial pressure	135 [126–140]	133 [125–147]	135 [130–145]	130 [125–140]	135 [130–145]	0.598
Admission congestion score	3.63 [±1.02]	3.53 [±1.05]	3.27 [±1.10]	3.13 [±1.05]	3.80 [±1.10]	<0.001
Discharge congestion score	1.15 [±1.17]	1.63 [±1.36]	1.11 [±1.17]	0.95 [±1.11]	1.55 [±1.38]	0.001
Admission serum creatinine (mg/dL)	1.03 [0.84–1.43]	1.60 [1.30–2.07]	1.73 [1.29–2.20]	1.25 [1.03–1.62]	1.23 [0.99–1.63]	<0.001
Discharge serum creatinine (mg/dL)	1.08 [0.90–1.41]	1.70 [1.40–2.24]	1.31 [1.00–1.70]	1.29 [1.00–1.73]	1.66 [1.27–2.26]	<0.001
Admission eGFR (mL/min/m^2^)	58 [46–77]	39 [32–53]	35 [25–51]	49 [36–64]	49 [38–66]	<0.001
Discharge eGFR (mL/min/m^2^)	61 [45–75]	35 [28–52]	52 [35–67]	49 [34–65]	37 [24–53]	<0.001
In–hospital IV mean daily furosemide dosage (mg/die)	120 [100–120]	125 [120–150]	125 [100–150]	100 [80–120]	120 [120–175]	<0.001
Admission NTproBNP (pg/mL)	6,720 [3,863–11,325]	6,440 [2,824–10,700]	7,230 [4,939–14,200]	7,545 [4,478–11,246]	7,520 [4,826–16,956]	0.366
Discharge NTproBNP (pg/mL)	3,110 [1,184–7,238]	3,147 [1,187 −9,687]	3,358 [1,853–5,790] 60.3	2,782 [998–6,135]	4,594 [2,100–7,126]	0.062
Previous CHF (%)	53.2	60.3	66.7	58.2	50.5	0.298
ICD (%)	12.7	14.0	11.1	5.1	18.7	0.076
**Home therapy (%)**						
Loop diuretics	50.0	70.2	71.4	56.1	50.5	0.007
ACEis/ARBs/ARNI	72.2	71.9	68.3	81.6	65.9	0.157
Beta Blockers	72.0	75.4	69.8	80.6	70.3	0.446
MRAs	9.5	17.5	4.8	6.1	12.1	0.098
Digoxin	5.7	17.5	6.3	2.0	12.0	0.003
Ivabradin	19.0	8.8	12.7	17.3	23.1	0.180
Death (%)	22.8	40.4	31.7	10.2	25.3	<0.001
Rehospitalization (%)	17.1	28.1	12.7	5.1	26.4	<0.001

*ACEis, Angiotensin Converting Enzyme Inhibitors; ARBs, Angiotensin receptor blockers; ARNI, Angiontensin Receptor Neprilysin Inhibitor; CV, Cardiovascular; CHF, Chronic heart failure; CKD, Chronic Kidney Disease; CAD, Coronary artery disease; eGFR, estimated Glomerular filtration rate; ICD, Implantable cardiac defibrillator; IV, Intravenous; IVC, Inferior Cave Vein; LVEDD, Center ventricular end-diastolic diameter; LVEF, Center ventricular ejection fraction; LVESD, Center ventricular end-systolic diameter; MRAs, Mineralocorticoid receptor antagonists; NTproBNP, Aminoterminal pro B-type natriuretic peptide; S, Stable; PASP, Pulmonary artery systolic pressure; PI, Permanent improvement; PW, Persistent worsening; RVEDD, Right ventricular end-diastolic diameter; TAPSE, Tricuspid anular plase systolic excursion; TI, Transient improvement; TW, Transient worsening*.

### Renal Trajectories and Congestion

Of the 467 recruited patients, 192 (41,1%) encountered the primary composite outcome defined as death or CV hospitalization during 180 days of follow up. Among these patients, 112 died and 80 were re-hospitalized due to CV causes. Patients with an adverse composite outcome had a higher percentage of congestion at discharge compared to events free patients (44.8 vs. 17.5%; *p* < 0.001). Similarly, patients with NTproBNP decrease at discharge <30% experienced an increased adverse events rate compared to those with NTproBNP decrease more than 30% (52.1 vs. 28.4%; *p* < 0.001). Among different RF trajectories, TI and PW groups demonstrated a significantly (*p* < 0.001) higher rate of death (40.4 and 25.3%, respectively) and re-hospitalization (28.1 and 26.4%, respectively) compared to other groups (Table 2).

### Composite Outcome

**Figure 2:**
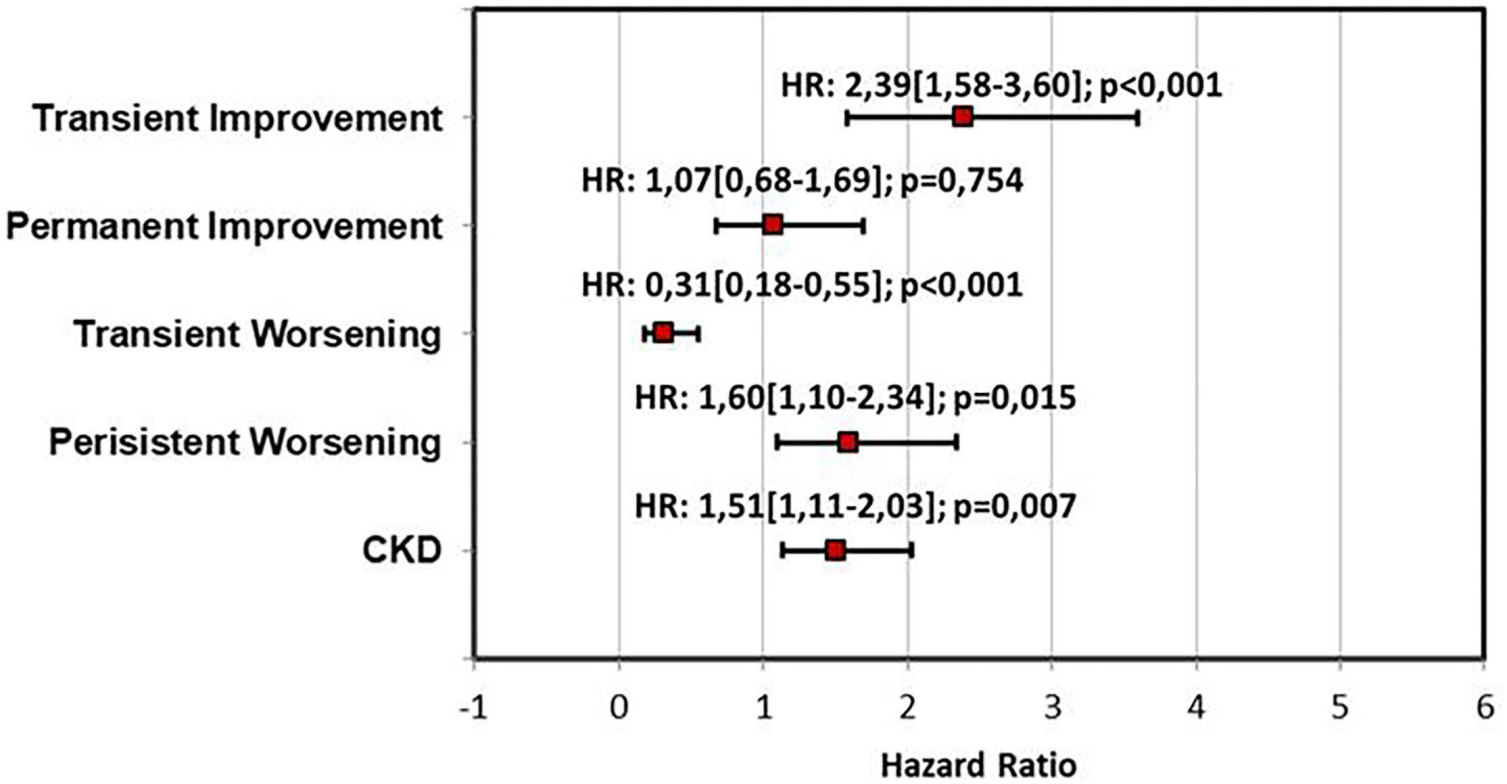
Multivariable analysis for outcome prediction including renal function patterns and CKD. CKD, Chronic Kidney Disease; HR, Hazard ratio.

**Figure 3:**
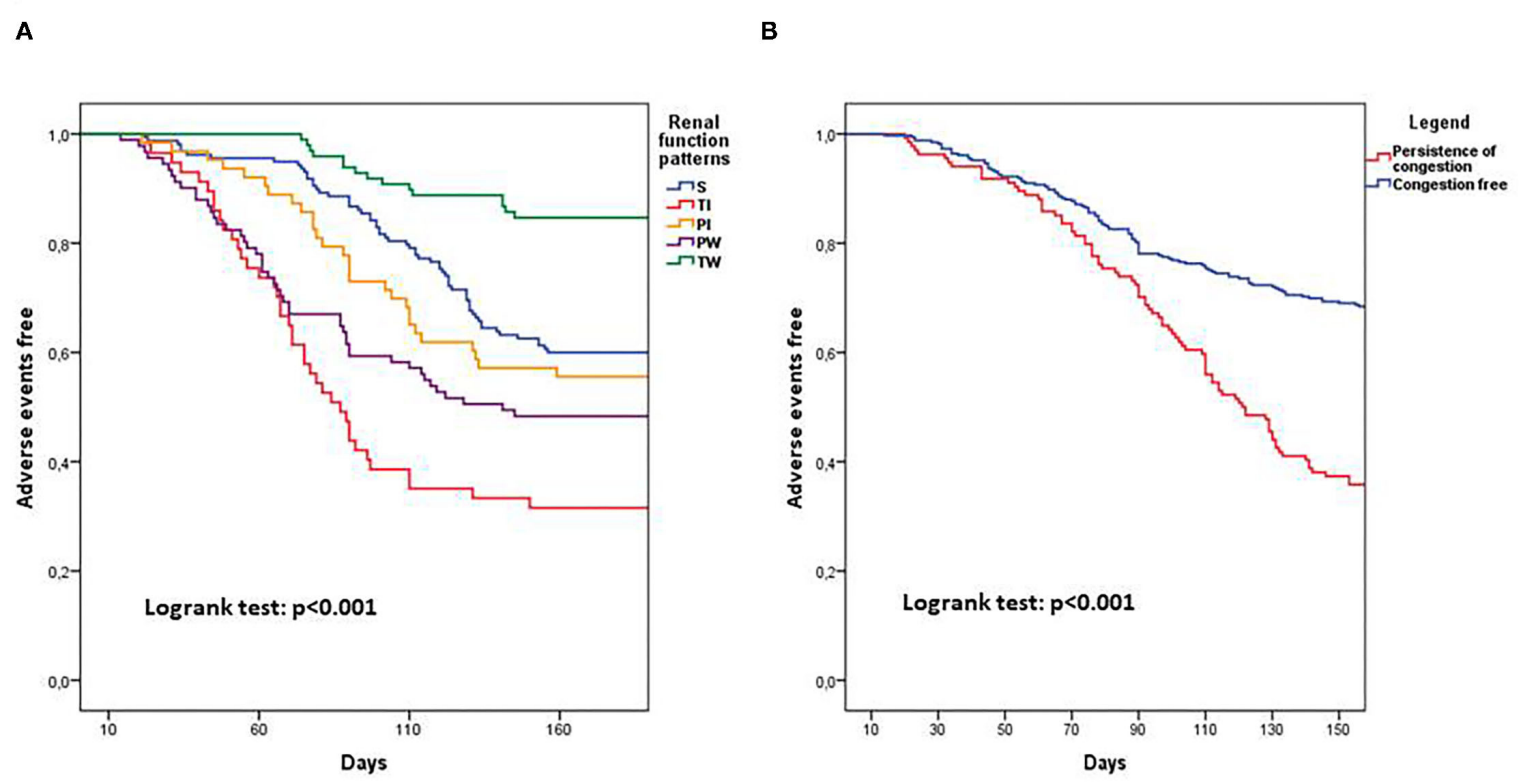
Kaplan Meier curves showing 180 days prognosis dividing patients for renal function patterns **(A)** and for persistence of clinical congestion **(B)**. PI, Permanent improvement; PW, Persistent worsening; S, Stable; TI, Transient improvement; TW, Transient worsening.

A univariate analysis of the renal function pattern demonstrated that the TI pattern was significantly related to poor prognosis [HR: 2.71 (1.81–4.05); *p* < 0.001] as was the PW pattern [HR: 1.68 (1.15–2.45); *p* = 0.007]. Conversely, the TW pattern showed a significantly protective effect on outcome [HR:0.34 (0.19–0.60); *p* < 0.001]. Persistence of congestion and BNP reduction ≥30% were significantly related to clinical outcome at univariate analysis [HR: 2.41 (1.81–3.21); *p* < 0.001 and HR:0.47 (0.35–0.67); *p* < 0.001]. Similarly, in the univariate analysis, CKD was related to poor prognosis [HR: 1.52 (1.14–2.04); *p* = 0.004; Table 3]. Multivariable analysis including renal function patterns and CKD confirmed the independent prognostic role of TI [HR: 2.39 (1.58–3.60); *p* < 0.001], TW [HR:0.31 (0.18–0.55); *p* < 0.001], PW [HR: 1.60 (1.10–2.34); *p* = 0.015] and CKD [HR: 1.51 (1.11–2,03]; *p* = 0.007] (Figure 2). A multivariable analysis combining renal function and persistence of congestion pattern confirmed the univariate analysis findings about TI [HR: 2.61 (1.75–3.91); *p* < 0.001], TW [HR:0.34 (0.20–0.60); *p* < 0.001], PW [HR: 1.52 (1.04–2.22); *p* = 0.032] and congestion persistence [HR: 2.29 (1.71–3.05); *p* < 0.001] (Table 3). Multivariable analysis including renal function patterns, persistence of congestion, BNP reduction ≥30% adjusted for age, gender, previous CHF, CKD, LVEF <50%, and CV risk factors confirmed the independent relation of TI [HR: 2.30 (1.52–3.50); *p* < 0.001], TW [HR:0.30 (0.17–0.55); *p* < 0.001], PW [HR: 1.51 (1.02–2.24); *p* = 0.04], persistence of congestion [HR: 1.87 (1.39–2.52); *p* < 0.001] and NTproBNP reduction≥30% [HR 0.65 (0.48–0.87); *p* = 0.004] with clinical outcome (Table 3). Kaplan Meier survival curves showed the significant relation among renal function trajectories and persistence of congestion with adverse events occurrence (*p* < 0.001) (Figure 3).

**Table 3:** Univariate and multivariable analysis for 180 days outcome prediction.

**Variables**	**Univariate HR [CI]**	***p*–value**	**Multivariable^a^ HR[CI]**	***p*–value**	**Multivariable^b^ HR[CI]**	***p*–value**
Renal function patterns						
TI	2.71 [1.81–4.05]	<0.001	2.61 [1.75–3.91]	<0.001	2.30 [1.52–3.50]	<0.001
PI	1.22 [0.78–1.91]	0.375	1.29 [0.83–2.02]	0.256	1.13 [0.71–1.80]	0.594
TW	0.34 [0.19–0.60]	<0.001	0.34 [0.20–0.60]	<0.001	0.30 [0.17–0.55]	<0.001
PW	1.68 [1.15–2.45]	0.007	1.52 [1.04–2.22]	0.032	1.51 [1.02–2.24]	0.040
S	Ref.	–	Ref	–	Ref	–
Persistence of congestion	2.41 [1.81–3.21]	<0.001	2.29 [1.71–3.05]	<0.001	1.87 [1.39–2.52]	<0.001
Δ NTproBNP reduction ≥ 30%	0.47 [0.35–0.67]	<0.001	–	–	0.65 [0.48–0.87]	0.004
CKD	1.52 [1.14–2.04]	0.004	–	–	1.33 [0.98–1.82]	0.067

a *Analysis including renal function trajectories and persistence of congestion*

b *Analysis adjusted for Age, Gender, previous CHF, LVEF <50% and CV risk factors*

*CKD, Chronic Kidney Disease; CI, Confidence Interval; HR, Hazard Ratio; NTproBNP, Aminoterminal pro B-type natriuretic peptide; PI, Permanent improvement; PW, P ersistent worsening; S, Stable; TI, Transient improvement; TW, Transient worsening*.

## Discussion

The current analysis has demonstrated that there are multiple subtypes of RF and each one likely comprises different mechanisms related to intrinsic kidney conditions, diuretic response, and congestion profile (24, 25). The study also explains some contrasting findings identified in the multiple analyses previously published, revealing a/the different impact of WRF. Indeed, the term WRF likely includes many patterns with different trajectories and specific significance (18, 19). Our results demonstrated that the TW group has a better outcome, similar to stable RF, whereas both permanent WRF and TI patterns showed a worse outcome. Our findings demonstrated that patients with permanent WRF and TI have a higher prevalence of unfavorable conditions, such as CAD, worse LVEF, and increased congestion, that could potentially impair outcome. Thus, the current findings may partially depend on adverse risk profile before admission. The recognition of different trajectories may be achieved only by repetitive blood sample measurements during the whole hospitalization period. Conversely, the simple evaluation of RF at admission and discharge as reported in most studies, is not sufficient to identify the real trend. A different RF pattern could be influenced by several features, such as the treatment adopted during the acute phase, baseline characteristics, presence of baseline CKD, and the congestion status (26). Notably, the far more dangerous pattern we identified needs to be contextualized according to the RF time course, CKD degree, systemic clinical conditions, haemodynamic status, and neurohormonal overdrive. Therefore, the intrinsic renal status may affect the different RF pattern. Hence, systemic blood pressure and kidney perfusion, increased central and renal venous pressure, tubulo glomerular feedback, and medullary and tubular state, are all potentially contributing factors (27, 28). Importantly, no universal agreement exists on when the renal blood marker must be measured in order to define and monitor RF in Acute decompensated Heart failure (ADHF) patients. Many trials have demonstrated that 30–40% of the patients hospitalized with HF experienced some WRF degrees (29, 30). However, not all studies agree about its prognostic role: an observational study showed that creatinine changes during hospitalization were independently associated with a higher risk of one-year mortality only in subjects with basal CKD (31). In the PROTECT Trial which included patients with some degree of CKD, many patients had a creatinine increase during hospitalization, and those experiencing more severe CKD showed a higher mortality during the 60 days follow-up (32). More recently, Holgado et al. demonstrated that AHF patients with more severe AKI degree had poorer prognosis (33). Interestingly, when WRF is associated with hemoconcentration or reduction in NTproBNP, it is not connected to an increased adverse event rate (34). Therefore, in a *post hoc* analysis of PROTECT in which serial measurements of RF were evaluated and defined in relation to specific trajectories, the authors did not find a remarkable difference among the different patterns (18). It is noteworthy that Testani et al., in a *post hoc* analysis, showed that patients with Improved Renal Function (IRF) had the worse prognosis compared with those with WRF (16). Similar findings have been recently replaced by Sai et al. and could be related to previous renal function deterioration before hospitalization or dynamic changes in central venous pressure during acute and post discharge phase (35). These contrasting findings suggest that RF should be evaluated during and after discharge to determine specific trends with their associated clinical characteristics and to detect those subtypes with increased risk (8).

Looking at congestion analysis, we showed that residual clinical congestion score before discharge is higher in permanent WRF and TI groups which are related to poor prognosis. Moreover, at multivariable analysis, the pre-discharge congestion is significantly related to adverse outcome. Although our findings may be influenced by the intravenous diuretic amount during the hospitalization period and population heterogeneity, they highlight the relevance of the concomitant measurements of hydro-saline retention and diuretic efficiency monitorization in the context of RF trajectories. Probably, in these groups, the persistence of congestion and in particular the presence of clinical signs of both pulmonary and peripheral congestion are the main driver of worse outcome but should be related also to diuretic resistance which was confirmed by the higher dosage of intravenous diuretics used during hospitalization (36). A proof of these theories appears to be confirmed by two *post hoc* analyses from PROTECT and RELAX-AHF trials, evaluating diuretic response during the hospitalization phase (37, 38). Accordingly, our data confirmed the strict relation among poor diuretic response, renal dysfunction, and congestion. Obviously, a multi-parametric assessment of congestion status through imaging integration of clinical congestion signs would have been most accurate in terms outcome prediction and fluid retention definition in this analysis (23). However, admission echocardiographic parameters such as pulmonary artery systolic pressures and inferior cave vein did not differ among groups as well as the index of left ventricle overload (E/e'). This finding of the current study confirmed the pivotal role of renal trajectories and clinical congestion in outcome prediction.

Interestingly, our analysis revealed that CKD was more prevalent in the groups with RF improvement (both transient and persistent). CKD severity appears related to adverse event occurrence, however, its impact may differ among different RF subtypes during hospitalization This data highlights the relevance of basal renal function status as one of the most important prognostic variables, and it suggests a need to look at both baseline RF and RF trajectories for better patient recognition. This appearance also confirms a previous metanalysis showing that CKD had a much greater unfavorable impact compared to WRF (39).

### Limitations

This is a retrospective, observational multicenter study conducted in three tertiary hospitals where patients admitted for AHF were usually older and with more comorbidities than those enrolled in interventional clinical trials. This item might explain the high rate of adverse events in terms of re-hospitalization and mortality during follow-up (34). Also, only consenting patients considered suitable for the DIUR-AHF trial were screened and enrolled in this study, which introduces further selection bias. However, with respect to similar studies, our analysis was not influenced by additional drug and study protocols because our sample was treated according to guideline recommendations. In addition, treating physicians were not blinded to the clinical congestion assessment and renal function modifications that occurred during hospitalization, and different therapeutic choices might have influenced results. The RF definition was arbitrary although it reflects another similar study, and therefore, in our estimation, the definition of WRF does not exactly match the recent classification that indicates deterioration over a longer period of time (5, 18). The RF trend may be influenced by the change in diuretic infusion amount and diuretic response that were not included in our analysis. Importantly, a comprehensive clinical evaluation might also take time, and the diuretic dosage amount and infusional timing period might have influenced congestion degree and renal patterns. Congestion evaluation has been performed by clinical assessment and NTproBNP values, and a more integrated study should comprise a detailed ultrasound evaluation by B-lines, cava vein, and peripheral impedance examinations. In this study, there is the lack of laboratory assessment of multi-organ and hepatic dysfunction which are usually a typical feature of patients with HF in the decompensation phases. Finally, our renal function patterns and screening were based on the in-hospital trend that reflect a relatively short observational period, and a longer evaluation, with 3- and 6-month creatinine values measurement after discharge, should help us to identify further renal fluctuations potentially responsible for different outcomes.

## Conclusions

Given the contrasting results regarding the prognostic relevance of different RF trajectories during AHF hospitalization, it becomes of paramount importance to identify the pattern with much more clinical relevance. The application of algorithms evaluating both different renal function changes and clinical congestion during the hospitalization period may help to distinguish subgroups with increased risk. Persistent deterioration and transient improvement appear to be the two patterns associated with increased risk. Further studies might be warranted to determine whether the contemporary assessment of these features could become an appropriate target for CRS-1 recognition and management.

## Data Availability Statement

Study data will be available after an official request to the corresponding author.

## Ethics Statement

The studies involving human participants were reviewed and approved by CEAVSE. The patients/participants provided their written informed consent to participate in this study.

## Author Contributions

APal: conception and design and drafting of the manuscript. FC: statistical analysis and support for data interpretation. APag, AR, LL, and AB: data collection and interpretation. MF: final approval of the manuscript and critical revision. NG: data curation and interpretation. GR: design and drafting of the manuscript, statistical analysis and support for data interpretation, and critical revision. All authors contributed to the article and approved the submitted version.

## Conflict of Interest

The authors declare that the research was conducted in the absence of any commercial or financial relationships that could be construed as a potential conflict of interest.

## Publisher's Note

All claims expressed in this article are solely those of the authors and do not necessarily represent those of their affiliated organizations, or those of the publisher, the editors and the reviewers. Any product that may be evaluated in this article, or claim that may be made by its manufacturer, is not guaranteed or endorsed by the publisher.

## Supplementary Material

Supplementary Figure 1 Study design of DIUR-AHF trial (20).

Supplementary Figure 2 Scheme of loop diuretic infusion in DIUR-AHF trial (20).

## Abbreviations

ADHF: Acute decompensated heart failure
AHF: Acute heart failure
AKI: Acute kidney injury
ACM: All-cause mortality
NT-proBNP: Amino terminal pro B-type natriuretic peptide
CRS-1: Cardio-renal syndrome type 1
CV: Cardiovascular
CAD: Coronary artery disease
CKD: Chronic kidney disease
eGFR: Estimated Glomerular filtration rate
HR: Hazard ratio
HF: Heart failure
IQR: Interquartile range
LVEF: Left ventricular ejection fraction
MDRD: Modification of Diet in Renal Disease
PI: Permanent improvement
PW: Persistent worsening
RF: Renal function
RD: Renal dysfunction
S: Stable
TI: Transient improvement
TW: Transient worsening
WRF: Worsening renal function.
