# Supplementary figures and images for: Different Renal Function Patterns in Patients With Acute Heart Failure: Relationship With Outcome and Congestion
Source: Front Cardiovasc Med. 2022 Mar 7;9:779828. doi: 10.3389/fcvm.2022.779828 (PMC8940261; doi:10.3389/fcvm.2022.779828)

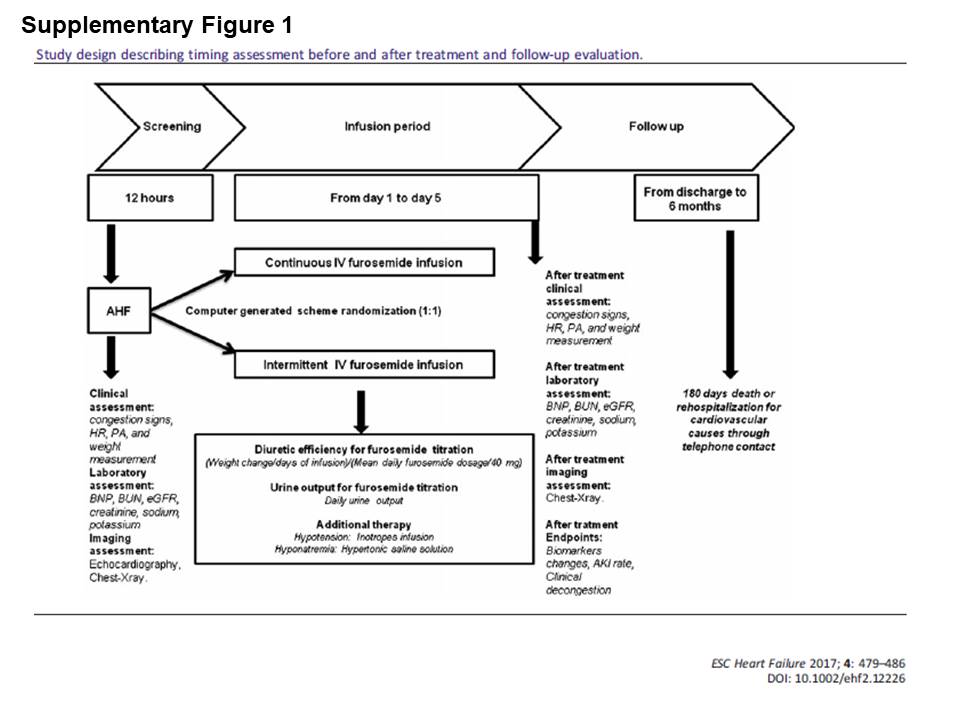

Supplement: Supplementary Figure 1 — Study design of DIUR-AHF trial (20). [file Image_1.jpeg]

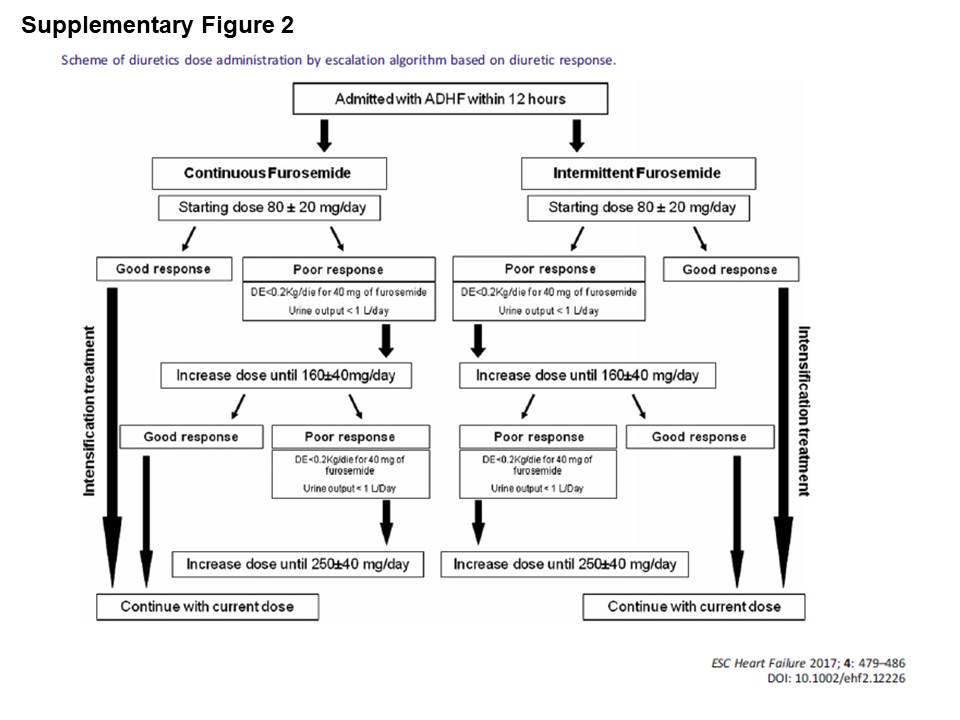

Supplement: Supplementary Figure 2 — Scheme of loop diuretic infusion in DIUR-AHF trial (20). [file Image_2.jpeg]
